# Complementing Online Data Dashboards with Generative AI to Enhance Gerontology Education

**DOI:** 10.1093/geroni/igaf122.1551

**Published:** 2025-12-31

**Authors:** Aaron Ogletree

**Affiliations:** Virginia Tech, Blacksburg, Virginia, United States

## Abstract

The universe of online data dashboards focused on presenting and visualizing population health data is rapidly growing. At the same time, educators are grappling with how best to use generative AI to support gerontology education. Despite the promise of these two innovative technologies for advancing the study of older adults, educators may be unsure about using them in practice. The goal of this presentation is to (a) introduce a range of online data dashboards that can be leveraged for the study of older adults, and (b) demonstrate how generative AI can support educators in using these dashboards to address gerontology competencies. We conducted a landscape analysis of data dashboards to identify those with information on older adults aged 65 and older. Focusing on a subset of dashboards, we then manually created a crosswalk between AGHE competencies for gerontology education and features available in those dashboards to develop curated (i.e., gerontologist drafted) classroom prompts. Separately, we used generative AI tools, like ChatGPT, to develop similar prompts. Comparison of manually curated classroom prompts with those originating from generative AI highlighted overlap between the two. That is, several of the manually curated prompts were also provided by generative AI tools. In some cases, the prompts originating from generative AI tools captured applications that we had not considered but that were nonetheless topical and relevant. Despite these strengths, limitations are noted. Our findings illustrate how educators in gerontology can leverage generative AI to teach about population health using online data dashboards.

